# Retroactivity in the Context of Modularly Structured Biomolecular Systems

**DOI:** 10.3389/fbioe.2015.00085

**Published:** 2015-06-17

**Authors:** Libertad Pantoja-Hernández, Juan Carlos Martínez-García

**Affiliations:** ^1^Laboratorio de Genética Molecular, Desarrollo y Evolución de Plantas, Instituto de Ecología, Universidad Nacional Autónoma de México, Mexico City, Mexico; ^2^Centro de Ciencias de Complejidad (C3), Universidad Nacional Autónoma de México, Mexico City, Mexico; ^3^Departamento de Control Automático, Centro de Investigación y de Estudios Avanzados del Instituto Politécnico Nacional (CINVESTAV-IPN), Mexico City, Mexico

**Keywords:** retroactivity, modularity, regulatory biomolecular networks, signal transduction, synthetic biology, systems biology

## Abstract

Synthetic biology has intensively promoted the technical implementation of modular strategies in the fabrication of biological devices. Modules are considered as networks of reactions. The behavior displayed by biomolecular systems results from the information processes carried out by the interconnection of the involved modules. However, in natural systems, module wiring is not a free-of-charge process; as a consequence of interconnection, a reactive phenomenon called *retroactivity* emerges. This phenomenon is characterized by signals that propagate from downstream modules (the modules that receive the incoming signals upon interconnection) to upstream ones (the modules that send the signals upon interconnection). Such retroactivity signals, depending of their strength, may change and sometimes even disrupt the behavior of modular biomolecular systems. Thus, analysis of retroactivity effects in natural biological and biosynthetic systems is crucial to achieve a deeper understanding of how this interconnection between functionally characterized modules takes place and how it impacts the overall behavior of the involved cell. By discussing the modules interconnection in natural and synthetic biomolecular systems, we propose that such systems should be considered as quasi-modular.

## 1 Introduction

The reuse of existing biomolecular modules and the construction of new ones with prescribed functionalities constitute obvious goals in the design of cellular biomolecular synthetic systems (here on, synthetic systems). As such, modular structure is a crucial and desirable organizational characteristic for them. Indeed, when designing a synthetic system in a modular way, we expect it to preserve the behavior (i.e., the functionality) of its modules upon interconnection. Modularity, as an engineering design strategy, is intended to limit both structural and functional complexity. However, given that biodevices (i.e., synthetic systems) are commonly embedded in pre-existing natural organisms, modularity is constrained by the functional limits imposed by them. One of the main topics discussed in the Section “Modularity” is the pertinence of considering biomolecular systems as modularly structured. We argue that biological systems – independently of the involved level of design – may not satisfy the functional dissociation and independence conditions required for being modular and may not fulfill as well the requirements to be considered fully interconnected (i.e., non-modular). Thus, we claim that biological systems should be considered “quasi-modular.”

**Figure 1:**
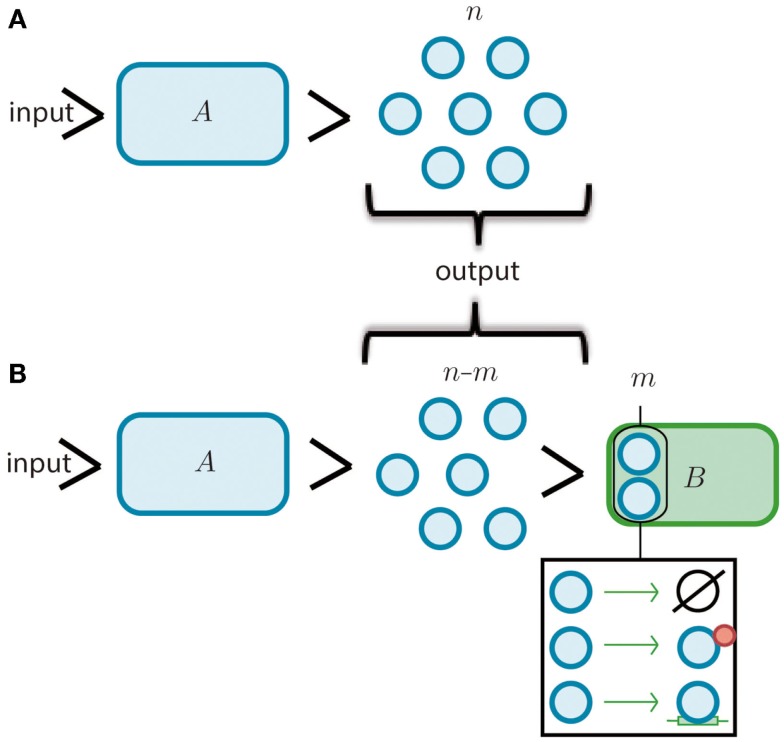
**Modules and retroactivity**. **(A)** A biomolecular module, *A* (whose given function is to produce a finite set of *n* molecules given an input signal, *i.e*., a *stimulus*). **(B)** The output of *A* is connected to the input channel of a second module, *B*. This connection will decrease the signal output of module *A* by *m* molecules that are degraded, sequestered or transformed by *B*. In the black square we include the options of degradation, covalent modification and simple sequestration by binding. If module *B* significantly changes the original signal coming from module *A* (*i.e*., imposing a load in its input channel), the functionality of this upstream module may be changed or even disrupted.

In biological systems, the term *retroactivity* [see Saez-Rodriguez et al. (2005) and Del Vecchio et al. (2008)], adapted from the well-known notion of *impedance* (or *load*) of analog electrical and electronic circuits, refers to a counter-responsive (i.e., reactive) molecular signal that arises from the interconnection of functional biomolecular modules. Retroactivity effects have been approached in the literature but under different names, such as “loads effects,” “tritiation,” etc. For a schematic illustration of the retroactivity concept, see Figure 1.

In this review, we focus our attention on the role played by retroactivity in modularly structured cellular biomolecular systems (here on biomolecular systems). We begin with the Section “Modularity,” where we present and give a brief explanation of some definitions of this concept. Then, we include a brief discussion of modular structuring in biological systems. Such discussion comprises the possible consequences of having very modular systems (i.e., systems where just a few biochemical components connect the functional modules) as well as those resulting from the complete lack of a modular structure. The latter means highly interconnected biomolecular components where each node participates in many tasks within biological systems, according to Alon (2003).

After reviewing modularity, we then focus the discussion on retroactivity in the equally titled section, where we thoroughly explain this concept and its associated definitions in biomolecular systems. Then, we summarize the historical progress in the study of this topic. We recall one of the original uses of retroactivity in the identification of modular structures in high-throughput data characterizing biomolecular networks. Thereafter, we further elaborate the role of retroactivity in biosynthetic constructs and why dynamic insulation could be required to ensure modularity in biomolecular networks. Additionally, we describe the possible use of biomolecular noise to calculate retroactivity. After that, we review modification cascades and kinetic insulation as possible natural mechanisms to address the effects of retroactivity. We finish this section with a discussion focused on the functional effects of retroactivity verified *in vitro* and *in vivo*.

In the section “Conclusions and Perspectives,” we do a brief recapitulation of the previous sections and share our thoughts in the forthcoming implications of retroactivity in both systems and synthetic biology.

## 2 Modularity

Defining modularity is a challenging task. A standard dictionary defines a module as “an independent unit that can be used to construct more complex structures.” However, there are not independent components in natural biomolecular systems. Molecules, complexes, and other structures are commonly shared across different pathways. Even the delimitations that bring about independence, to some extent, are highly dynamic and may change the system’s structure at any time.

The existence of multiple descriptions of modularity in biological sciences, ranging from those related to organisms’ morphology to those dealing with functional biomolecular aspects [as discussed in Kim et al. (2011)], may be at the origin of the struggle to establish a consensual definition of modularity in biomolecular systems. We proceed to mention some definitions of modularity that, in our opinion, are helpful in defining a conceptual frame to gain a deeper understanding of retroactivity in biological systems.

In Hartwell (1999), the author considers that functional modules are composed of many different kinds of interacting molecules that give rise to specific discrete functions. These functions cannot be easily predicted from the study of the module’s components in isolation. He also considers that modules are a critical level of biological organization.

A definition of functional module that complements Hartwell’s idea is “features that act together in performing some discrete physiological function and are semi-autonomous in relation to other functional modules” [see Wagner et al. (2007)]. This, along with the criteria that define a module introduced in Saez-Rodriguez et al. (2004) – common physiological task, common genetic units, common signal transduction network – provide a conceptual background where considering molecular complex systems as modular may help to understand the properties that emerge when interconnected modules.

We would define a module bearing in mind that they are “parts of a system that have semi-independence; in the sense that the ties within them are stronger than any other ties with other components not belonging to the module” [see Rasskin-Gutman (2005)]. We also consider that a given biomolecular module would have an associated function (or behavior) and that a biomolecular module is an input/output dynamical system. Under this operative definition, the relationships are determined by physical interactions between molecules. Depending on its functionality, their parameters can be expressed as: dissociation constants, association or dissociation rates, and rates of change (production or degradation), among others. The strength of the interaction is related to the parameter value (intensity) and the function persistence.

As far as bioengineering is concerned, although shaping a biosynthetic system in a modular way is clearly advantageous, it is a complicated task. In an ideal scenario, when constructing a synthetic system, parts would be interchangeable. In that ideal case, the system would be easy to maintain by renewing its parts and replacing any one prone to failure. Under this scheme, the parts could be integrated and interchanged in different ways to achieve new functions.

In Endy (2005), the author stresses the main challenges limiting the development of synthetic biology. Among them, he mentions “an inability to avoid or manage biological complexity” and “the tedious and unreliable construction and characterization of synthetic biological systems.” He suggests considering lessons from engineering, which could be valuable for synthetic biology nowadays. He mentions that, from his point of view, the three ideas most relevant for synthetic biology may be standardization, decoupling, and abstraction. The decoupling idea is intimately related to the capability of defining independent modules, which could work separately or coupled. Referring to standardization, the definition of a standard bio-part may need the consideration of retroactivity in order to define independent parts or to include standard insulators as part of the synthetic biologists toolbox.

Another work relating modularity with synthetic biology is (Andrianantoandro et al., 2006) where the authors emphasize the role of modules in synthetic biology: “At the module layer, the synthetic biologist uses a diverse library of biological devices to assemble complex pathways that function like integrated circuits. The connection of these modules to each other and their integration into host cells allows the synthetic biologist to extend or modify the behavior of cells in a programmatic fashion.” They also emphasize how modules are embedded in host cells, which they modify and by which they are modified themselves. We will come back to this topic later.

In Alon (2003) and the references therein, modularity is proposed to be a necessary structural property for natural biomolecular systems. The author stresses that modularity promotes dynamical features such as robustness to component tolerances, easy reconfiguration for new stresses or conditions in the short term, and capacity to test functions in the long term (evolution). Given that in natural systems biochemical components participate in multiple pathways, we favor a non-absolute posture defending that biomolecular systems are *quasi*-modular. This is a claim that should be taken with some reserve. In the next part of this section, we intend to present some evidence underlying this statement.

In Sriram et al. (2009) and Benítez and Álvarez Buylla (2010), the coupling of extra components is shown to provide robustness to the involved biological systems without affecting the dynamical steady-states that they may achieve. This is an example of how a module may need more connections, other than the context where it is embedded, in order to be fully functional. The same function – reaching a specific set of dynamic attractors – can be obtained by a certain set of elements and connections (a functional biomolecular module) regardless of the redundancy of the modules or feedback loops.

In Benítez and Álvarez Buylla (2010), any of the feedback loops could be considered as a functional module. The complexity of networks studied in that work is further simplified by feedback loops that minimize the number of independent (or *quasi-independent*) components (modules) in the network. Thus, while the input and output of the system are conserved regardless of the subtraction or incorporation of feedback loops, the loops and redundant elements provide dynamical diversity to the system (i.e., stability, robustness, etc).

In natural biomolecular systems, the constant inter-linkage between heterogeneous parts makes difficult to establish the structural and functional boundaries that delimit modules. These heterogeneous parts include covalent modification enzymes and transcription factors, among other compounds; they participate in multiple pathways, intertwining the functions in such a way that they might not be modular. An absolutely modular system would be one composed by a given set of distinctive cooperative functional subsystems (i.e., modules) or one with well-separable components. In an extreme case, we would take each single biomolecule as a module. Out of this obvious and uninteresting case, we are concerned by systems understood as networks of chemical reactions, described as systems structured in hierarchical connections of discernible functional components. Natural systems do not have all their biochemical components connected with each other, as would happen if they were totally connected constituting a single module. The former may be obvious for the reader familiar with integrative approaches in transcriptional regulation and signaling. However, we want to further emphasize that biological systems seem to strike a balance between modularity and full connectivity. Even if they have physical and chemical isolation to a certain extent, such isolation is usually “broken” selectively allowing a level of integration that is more intimate than would be expected in a modular system. This integration seems to be achieved by redundancy, feedback, or interconnection effects.

Even if modularity *per se* does not make better the system’s robustness to parameter changes or fluctuations, it does improve the management of mechanisms that address local problems in the network. Modular structures therefore provide functional resilience, a necessary requirement for both robustness and plasticity issues.

**Figure 2:**
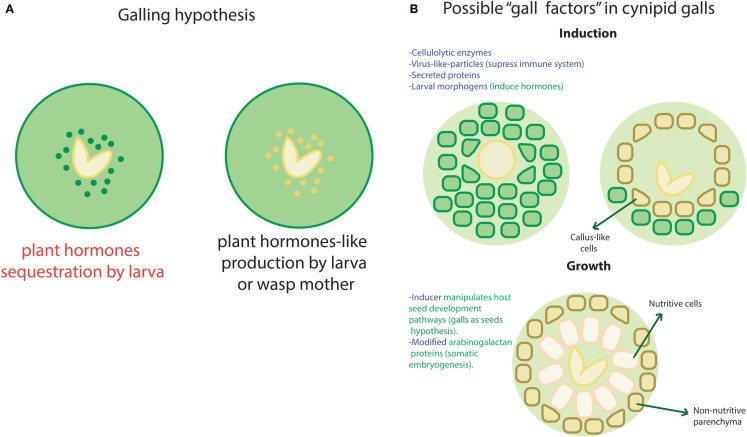
**Galling hypothesis**. A few hypothesis about how galls take place and maintain in plants have been posed. This image intends to summarize most of them according to the exposed in Hearn (2014) and Tooker and Helms (2014). **(A)** Regarding hormones, two likely ways have been proposed to induce and maintain a gall. In the first one, the wasp larva could be sequestering hormones as in the left case (in red) or produce hormones-like metabolites that trick plant’s network as in the right case. **(B)** There are multiple factors in both wasp (blue font) and plants (green font) that could be involved in the induction and/or preservation of galls without the need to interfere directly with the hormones. Here we show some of such hypotetical factors in two phases of gall development: induction and growth. Changes in the plant cells and structures are indicated with arrows.

As we just mentioned, a biomolecular module may be defined as a set of components that are necessary to accomplish a well-defined independent function. However, at the transcriptional level, such modules would require a shared transcription machinery, which belongs to the non-modular part of the system. In the other hand, very modular structures contain interconnection nodes where external signals could enter the network, interfering with its normal functionality in the absence of well-defined ports and zealous border guards. Thus, the dominance of modular structures in a network may allow easy “invasion” by external stimuli. By “invasion,” we imply undesired regulation by the interception of the modules’ input ports. Such disruptive entries may come from viruses, horizontal transfer, conjugation (in the case of bacteria), and even normal fluctuations in molecular concentrations. The disruption could be caused by any molecule suited to crosstalk with the module entry points, for example, second metabolites, transcription factors, or small RNAs. Given the former, absolute modularity might affect the native system’s controllability^1^ with probable behavioral undesirable consequences [see, for instance, Liu et al. (2011)]. Insect galls provide a dramatic example of how functional disruption takes place when there is an open port. Insect galls are structures that plants develop upon infestation by certain insect species, commonly shaped as fruits or other plant structures. Even if the involved circuitry and morphogenetic pathways of these structures remain obscure, it is clear that the insects are able to induce a complex goal-oriented and well-localized differentiation process with a minimal amount of input signals [see, for reference, Raman (2011)]. Diverse hypothesis have been posed regarding how this structures are induced and maintained. In the following, we mention some of the ones that have been posed specifically for cynipid wasps. One of them suggests that plant hormones as auxin may be sequestered by the larva as shown in Figure 2A. However, the high concentrations found in such structures have led to consider this hypothesis unlikely [see Straka et al. (2010) and Tooker and Helms (2014)]. Recent evidence suggests that some insects, as saw fly, are able to produce their own versions of plant hormones as shown in Figure 2A [see Yamaguchi et al. (2012) and Tooker and Helms (2014)] and thus control plant morphogenesis. The thesis by Jack Hearn makes a very complete review of the current knowledge regarding cynipid wasps [see Hearn (2014)]. We enlist some of the proposed inputs that could have some impact in the plant pathways recruiting by the wasp larva in Figure 2B. This shows that, even if the exact path for this particular form of infection remains elusive, there are multiple entries that could be used (alone or in combination) to achieve control of a host network. The previous statements reflect the inner fragility of some biological networks that may be caused, at least partially, by its modular structure.

Let us consider again the role of shared resources. In the case of synthetic systems, basal machineries may be considered independent of the module to embed as they would be part of the chassis. Under this vision, an independent module would be functional provided that an input and the adequate basal machinery are present. We are aware that under different contexts (e.g., stages of development, living styles, or environmental conditions, among others), the machinery may be changed as even the chassis can be subjected to stress. For natural biological systems, the machinery and chassis would take part in the module’s embedding and its connection to the rest of the system as has been proposed with a queuing theory formalism in Cookson et al. (2011). The authors propose that proteins are “queued” before being degraded by proteases. Their place in the queue depends on the abundance of other proteins (which is an example of intertwining) to be degraded. We could summarize what we previously intended to assert in a statement made in Jayanthi et al. (2013). The authors argue that the competition for resources between modules breaks modularity.

Given the previous reasons, we propose to consider biological systems as *quasi*-modular, i.e., systems where feedback; multiple inputs connecting the module intermodularly; and redundancy, decrease the modules’ independence. This means that, when studying biomolecular networks, there are clusters of components devoted to tasks (those tasks can be more than one). These clusters are connected through outputs between them, but not all the components are necessarily connected with each other. This is further complicated by the dynamic change in the collection of biomolecular components that contribute to a specific function. The latter reinforces the variability that underlies the modules’ behavior.

*Quasi*-modularity is a survival strategy, because preserving functions under different conditions is imperative for life. In biological systems, random changes (e.g., mutations) typically do not cause the rupture of the entire system. Similarly, in most of the cases, the disruption of one module would not mean the same for the whole system. From this perspective, we could say that in an ideal scenario, natural biological systems might be just modular enough to avoid sustained external invasion. This may be an adventured statement as we have also provided an example where some co-evolved parasites can access host systems through interfering pathways. However, it is expected that biomolecular systems will tend to have few input entries in order to reduce such invasion possibility. Here, we consider that the module is formed solely by the core components capable of directing the module’s function when embedded in an adequate background. We choose to define it this way, because the intricate regulation of natural systems may need more than one regulatory element devoted to preserve and use the original function of the module (in terms of its output molecules) and because in this way, *quasi*-modules are better suited to synthetic biology conception.

The analysis of different types of complex networks in light of John Rhodes’ *mini-module* analysis^2^ has shown that sometimes overall system complexity may be reduced by new connections, as discussed in Rhodes (2010). In that work, the complexity of a given dynamical system is a measure of its minimum amount of constitutive mini-modules with an input and an output. This provides an illustrative example of the interplay between biological complexity and modularity.

Due to the scope and space limits, we could not make reference to all the works we would like to. However, we invite the reader to check the following publications regarding modularity in biological systems in relation with epistasis, networks, and evolution: (Raff and Raff, 2000; Hansen, 2003; Segre et al., 2005; Lorenz et al., 2011; Ten Tusscher and Hogeweg, 2011; Clune et al., 2013; Babu et al., 2014).

In what follows, we review the historical process of the study of retroactivity signals as well as their role in biomolecular systems.

## 3 Retroactivity

A modular biomolecular network can be seen as an information processor, where the flux is expected to be reliable and functionally immutable to interconnections. However, connection between modules is not always free of charge. Indeed, when a given module is connected downstream to a second one, a reactive phenomenon known as *retroactivity* arises. In this connection scheme, the output channel of the first module is connected to the input channel of the second one, thus guaranteeing that the flux will go from the first to the second module. Retroactivity is defined in Del Vecchio (2011) as “the phenomena by which, the behavior of an upstream system is changed upon the interconnection of a downstream one.” Thus, retroactivity comprehends all the interconnection consequences previously identified as loads effects, molecular tritiation, and squelching to name a few examples. We can interpret retroactivity as a toll that must be paid by modularly structured systems, such as the engineered ones, in order to ensure information flow. Mathematically, retroactivity can be represented as the terms that arise in the rate of change of the upstream system’s output upon interconnection. For further clarification, please refer to Box 1.

Box 1 **Isolated versus connected system.**

Biomolecular systems can be modeled by considering them unidirectional networks of independent elements, rarely taking into account the retroactivity signals involved in the interconnections (Figure 5A). Networks inferred by high throughput data are typically this way (or even undirected) because of the lack of enough information. To exemplify the signal emergence of retroactivity, we consider a functional module embedded in a simple given chain of reactions. Connected to the embedded module, we have an upstream module and a downstream one. We represent the isolated module (i.e., the given module before embedding it within the reactions chain) as *S* in Figure 5A. When embedding module *S* into the chain, two new reactive signals appear, signal *r* and signal *s*, as shown in Figure 5B. As a result of the interconnections, the given module *S* is now connected upstream to the *S_i_* module and downstream to the *S*_0_ module as shown in Figure 5C. The signal *r* then flows from the given module *S* to the downstream module *S_i_*, and the signal *s* flows from the downstream module *S_0_* to the module *S*. Both reactive signals (*r* and *s*) flow in the opposite direction of the main signal flux involving signals *z* and *x*, which characterize information processing, as shown in Figure 5C. Retroactivity formalization can be further elaborated with an example concerning a simplified gene regulatory process as follows: given a gene *X*, for a transcriptional module, its input signal is the time-dependent concentration of the transcription factor that regulates *X*, and its output signal is the corresponding concentration of free protein *x* into the cell. Here, transcription factors act as inputs, while protein concentrations of their downstream regulated genes (which can also be transcription factors) act as outputs [see Alon (2007) and Del Vecchio (2011)]. This means that the output of a module could be the input signal for a downstream module. The corresponding change in the amount of *x* depends on its production rate [denoted *k*(*t*)] and its degradation rate *δx*, with *δ* standing for the constant degradation rate. In an isolated case, the dynamics of *x* can be expressed in simple continuous-time terms in the following way:

$$\frac{\text{d}x}{\text{d}t}=k\left(t\right)-\delta x.$$

We shall restrict our further exposition to the discussion of retroactivity to the output (*i.e*., that related to the output signal and downstream module as the *r* signal shown in Figure 5C). A process of binding and unbinding takes place when the second module becomes regulated by the first one, forming a network constituted by the connection of two transcriptional modules (see Figure 6). This process is controlled by the ratio of the dissociation rate versus the association rate. Thus, the rates of association and dissociation of *x* to the promoter *p* are crucial for the retroactivity effects on the concentration of tx. Such rates are commonly denoted by *k*_off_ and *k*_on_, dissociation and association, respectively. The rate of change of the DNA-*x* complex can be written in an explicit and simplified way in ordinary differential equations [see Del Vecchio et al. (2008)]: 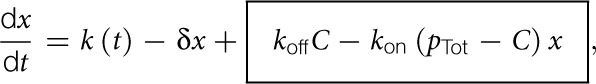

$$\begin{array}{ll} & \frac{\text{d}C}{\text{d}t}=-k_{\text{off}}C+k_{\text{on}}\left(p_{\text{Tot}}-C\right)x, \end{array}$$

where *C* denotes the concentration of the complex between *x* and the promoter, and *p*_Tot_ is the total concentration of promoter (free plus complex).

In the practice, retroactivity may be unimportant and leave the systems functionalities unchanged. However, depending on the parameters and the structure of the connection, retroactivity can generate ultrasensitive responses, cause a delay in reaching the expected steady state, or even disrupt it.

The functional implications of retroactivity may be hard to unveil. The input/output functionality of an isolated module defines its external “normal behavior.” If not attenuated, the retroactivity signal may act as a disturbance to the upstream module, changing that module’s “normal behavior.” For natural biological systems, most of these characteristics and functionalities may be unclear because retroactivity signals are integrated in natural systems’ circuitry, where they could be shaping outputs, or even compartmentalizing responses in time and space. The recent resurgence of topics related to interconnection effects provides an opportunity to make the most of the mathematical and computational formalizations of this phenomenon. These formalizations may shed further light into the properties of previously studied motifs and patterns of interconnection under the light of their capacity to minimize or potentiate retroactivity.

**Figure 3:**
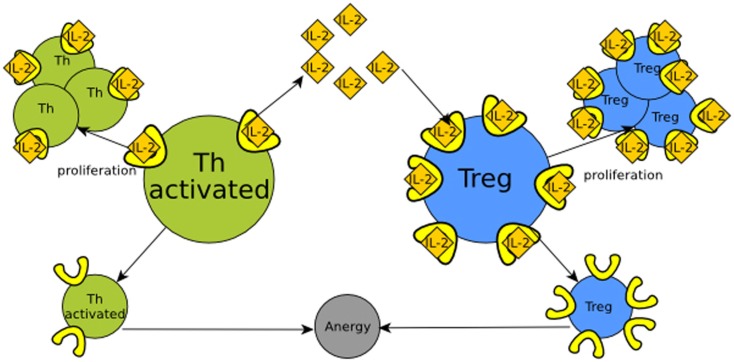
**Regulation of Th (T helper) cells (in green) by retroactivity**. Th cells produce IL-2 (yellow tangerine diamonds), which is sensed by receptors on their own cell surface (in bright yellow), resulting in proliferation. Th cell would be equitable to module *A* in Figure 1 and IL-2 to its output, which in this particular example is also its own input. If there is not enough IL-2, the cells become anergic and rapidly die. T regulatory cells (Treg, in blue) also have IL-2 receptors. This makes Treg cells capable of sequestering IL-2 and then analogous to the *B* module in Figure 1. The retroactivity and loads effects become significant as T regulatory cells have more receptors than Th cells, which enables them to regulate by competition with the Th population.

IL-2 (interleukin 2) signaling provides an example of the functional role of retroactivity regulation in immune cell populations. In the presence of an immunological challenge, T helper (Th) cells produce IL-2. When this molecule binds/activates the IL-2 receptor (IL-2R), Th cells proliferate (Höfer et al., 2012). If there is not enough IL-2, Th cells enter a state called anergy and die. T helper regulatory cells (Tregs) also require IL-2 in order to proliferate, and overexpress the high-affinity IL-2 receptor. Because of the aforementioned disparity in the number of IL-2 receptors and affinity, at a given quantity, Tregs may hijack the free IL-2 molecules, inducing their own proliferation. This reduces the quantity of IL-2 available, which may induce anergy in Th cells. Hence, Tregs control the population of Th cells by means of retroactivity. Here, retroactivity becomes relevant as the regulatory function depends on the interconnection parameters that give rise to retroactivity such as binding rates, IL-2 degradation, and population sizes. See Figure 3 and Box 1 for further clarification.

It is still under discussion whether biological systems tend to minimize retroactivity; use it as a way to shape signals or if they can potentially perform both functions and display them selectively depending on the context. We must point out that, in mathematical systems theory terms, it is well known that the interconnection of two different systems requires the internal state variable of both systems to be disjoint. This observation has been essential in further studies involving retroactivity and modularity concepts in synthetic biology as in Kim and Sauro (2010).

Now, we are going to review some of the current research on retroactivity and, in this context, we recall the original motivation underlying retroactivity research (the already mentioned identification of modular structures in high-throughput data characterizing biomolecular networks). Then, we focus on the key role that retroactivity plays when considering the implementation of biosynthetic modular constructs and why dynamic insulation is required to ensure modularity in biomolecular networks. Another mentioned topic is the role that noise can play in retroactivity measurement in *wet lab* approaches. We finish this section by reviewing kinetic insulation, the functional effects of retroactivity verified *in vitro* and *in vivo*, and some current technological applications of synthetic biology involving the manipulation of retroactivity.

### 3.1 Network modularity and retroactivity research

Modular organization of biomolecular networks would imply the existence of strong interdependencies between discrete sets of biomolecular components. Therefore, the possibility of finding functional biomolecular modules by analyzing data generated by high-throughput methods caught the attention of the scientific community at the start of the “–omics” revolution [see Hartwell (1999) and Thompson et al. (2002)].

Some observations, such as whole genome duplications and genomes’ modular structures, may have contributed to the interest in biomolecular systems’ modularity. Starting with clustering techniques, the ideas and methods have been slowly refined as in Bertone and Gerstein (2001) and Kim et al. (2011).

**Figure 4:**
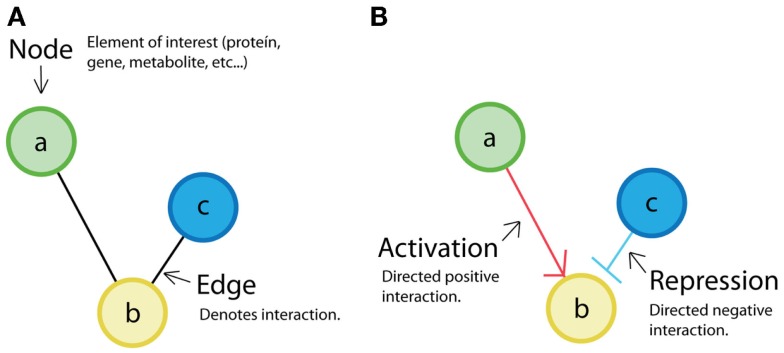
**Network representation: (A) classical representation of a non-directed network where nodes (a, b, c) are the elements of interest, and the edges represent interaction between elements**. **(B)** Example of a directed network. In directed networks, the order (from source to destiny) is evident. Sometimes, positive relations (activations) are depicted by arrows, and negative ones (repression) are depicted by truncated lines. It is also possible to find directed networks in which all relations are represented by arrows.

The retroactivity concept was introduced to biological systems’ research by Saez-Rodriguez et al. (2005). The authors proposed that modules in cell signal transduction networks can be defined by minimal retroactivity interactions. They considered that obtaining independent functional units would be interesting from a system-theoretical point of view. For this purpose, they required input-to-output unidirectionality of cell biomolecular modules based on network representation concepts (see Figure 4A).

This first view of retroactivity in the context of biology was biased by the possibility of making simplified coarse grain models of interacting modules. They aimed to use minimal retroactivity as their module-defining criterion.

Although the definition of module as “a group of nodes displaying minimal retroactivity” is useful for finding modules in networks, it implies that the components providing robustness and signal stability to the module are a part of it. This is at odds with our current module conception, but could be reconciled with it by a further dissection of the obtained modules.

Retroactivity-free reactions promote the existence of modular structures. Given the previous statement, in Saez-Rodriguez et al. (2005), the authors define their modules assuming that the interconnections between them are free of retroactivity. In a later work, Andrec et al. (2005) analyze how nodes are topologically connected in a network using a method called “Modular Responses Analysis” (MRA) to investigate what the particularities of the directionality of interactions between components are (see Figure 4B). Even if this was a good approximation, now it is known that due to the presence of indirect signals such as retroactivity, the MRA approach can be misleading. More recently, Sontag (2010) proposed a computational criterion to discover steady-state retroactivity and compare it to MRA. This is an alternative method for exactly detecting retroactivity signals that contrasts with a previous work by Kholodenko et al. (2002), where they proposed to identify functional interactions by perturbing modules in a network and measuring only global responses. In Kholodenko et al. (2002), they get an “interaction map” that can be expressed as modules’ interconnection strengths. However, this method also fails to deal with retroactivity. The inclusion of retroactivity in Sontag (2010) makes such network strategies closer to the actual biological systems.

Some references focused on the study of the interplay between loads and modularity are suggested in Saez-Rodriguez et al. (2008). A more recent discussion on this topic is posed by Alexander et al. (2009). they make an important statement: “knowledge of network structure is often not sufficient to infer function, and dynamical modularity can exist in the absence of structural modularity.” This is crucial for our further discussion, because it brings about questions regarding how natural biological systems establish boundaries, which regulatory circuits are involved in them and how they aid the dynamical remodeling of the biological systems’ networks. This is reinforced in some of the works cited by Alexander et al. (2009), such as the one by Ingram et al. (2006), where they show that important features of the network are transient and condition specific. Moreover, as also suggested by Alexander et al. (2009), transcription factors may act or not as hubs depending upon specific contexts. This has important implications for the role of retroactivity in natural biomolecular systems, because it means that the strength and function of retroactive signals could change dynamically. For example, nucleosomes could restrict the access of a transcription factor to downstream regulated targets and thus “liberate” an amount of transcription factor molecules from binding. Such nucleosomes could change their compaction level depending on the context. Another detail related to this discussion, and considered in the above publications, is that even network motifs have a wide range of possible behaviors depending on their associated parameters.

When designing synthetic systems in a modular way, it is of paramount importance to take into account retroactivity. In the next section, we provide some examples of how retroactivity is being considered in the context of current synthetic biology.

### 3.2 Rational design of biomolecular synthetic modules requires consideration of retroactivity

Before the concept of retroactivity was consolidated in biology, several ideas regarding the detrimental effects that exogenous elements may have in biomolecular systems were posed. For example, the squelching concept introduced in Gill and Ptashne (1988) and Natesan et al. (1997) refers to the possibility that large amounts of regulatory elements may deplete the cell’s resources, leading to a reduction of the system’s performance. A similar event occurs when enzymatic degradation queuing takes place. In that case, as we mentioned before, all the species of proteins that are degraded by a specific protease, become less likely to be degraded when there are high amounts of a given target protein. This happens because they compete for the protease [see Cookson et al. (2011)]. Molecular titration is another concept closely related to retroactivity that started to be studied almost simultaneously with the early formalization of retroactivity as in Buchler and Louis (2008) and Buchler and Cross (2009).

The presence of “non-functional” transcription factor-binding sites as loads in biomolecular systems remains an intriguing issue. They are ubiquitous in genomes, but their role remains obscure. However, in Burger et al. (2010), the authors suggest that such sites may be protecting transcription factor molecules from degradation. They also note some possible detrimental effects that the binding may cause depending on how the complex between the transcription factor and the binding site is degraded.

We recognize two main working branches of retroactivity in synthetic system developments. The first derived from the seminal Del Vecchio, Ninfa, and Sontag works and the second related to the Kim and Sauro research. Both branches have made important conceptual contributions to the recognition of retroactivity as an issue in synthetic systems design [see Del Vecchio et al. (2008), Sontag (2010), and Kim et al. (2011)].

In synthetic systems, modules are expected to be input–output systems where certain interconnection patterns achieve prescribed behaviors. Such modules would be expected to preserve their function upon interconnection. This is important when the retroactivity signal is performing a leeching role and is not intended to be used as a regulatory component of the system.

**Figure 5:**
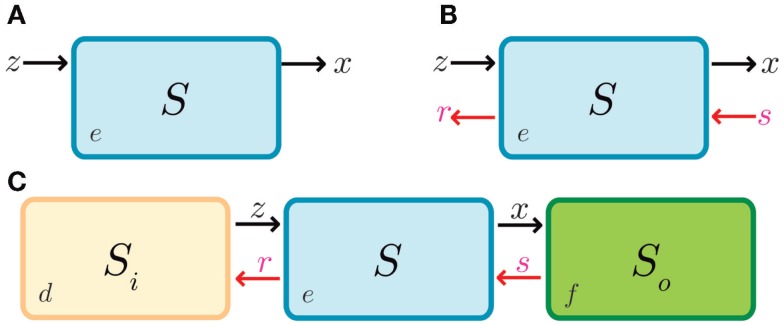
**Retroactivity in a box**. **(A)** Symbolic representation of the isolated input–output system as in Del Vecchio et al. (2008) and as an input–output system where retroactivity is neglected. *S* denotes the system, *e* is the system’s internal state, *z* is the input signal received by the module *S*, and *x* is the output signal emitted by *S* (which in this case coincides with the internal state of the system, *i.e*., *e*). Arrows indicate the signal direction as in Figure 2 (the functionality of *S* in isolation consists of processing signal *z* to get signal *x*). This figure is equitable to Figure 1A. Here, *z* would be the input and *x* the output. **(B)** Representation of the different signals that may be involved with *S* as a result of the interconnection; signals *r* and *s* correspond to retroactivity to the input and output, respectively. **(C)** Representation of the embedded system explicitly showing the upstream and downstream modules as well as the interconnection signals (*d* and *f* denote the internal state of system *S_i_* and system *S*_0_, respectively). Here, relative to *S*_0_, *S* would be analogous to *A* in Figure 1, and *S*_0_ would be equitable to *B* in Figure 1B.

The approach to retroactivity in Del Vecchio et al. (2008) is focused on systems dynamics. Their goal is to mathematically define and characterize the effects of retroactivity within systems and synthetic biology frameworks. In their first reports, they started from the following considerations: given a system (i.e., a module) that we call **S** with internal state *e*, where an associated input channel and an associated output channel are considered (which allows signal flux); a retroactivity signal may arise when the output channel of the system is connected to the input channel of another one. Two more terms are then aggregated to the original dynamics (see Figure 5A), one to denote the retroactivity to the output and one to denote the retroactivity to the input (see Figure 5B). With such tools, a formal definition and a measure for retroactivity were proposed when considering systems composed by chains of transcriptional modules. As shown in Box 1, several facts were elucidated by modeling the system under different operational conditions. In transcriptional systems, an amount of transcription factor equal or comparable to the number of their corresponding binding sites or a high affinity for such binding sites is indicative of a large retroactivity signal. As can be concluded from the analysis of the chain of two transcriptional modules, the retroactivity signal [i.e., *k*_off_*C* − *k*_on_(*p*_Tot_ − *C*)*x*, shown in Box 1] can be attenuated through fine-tuning some convenient parameters. However, the retroactivity signal depends on the time evolution of the concentration of *C* and *x*. This fact requires retroactivity attenuation to be attained via a robust dynamical scheme, i.e., through a dynamical insulator.

Kim and Sauro (2010) proposed the number of connections that a module can bear without a significant delay or attenuation (i.e., the fan-out) as a practical measure to quantify retroactivity. They considered that when two synthetic gene circuits are connected, it is usually by the regulatory mediation of transcription factors. They called module interface processes (MIPs) to all the processes involving the transcription factors as: transcription, translation, degradation, and the regulation of sites in the downstream module. Then, they mapped the MIPs into the model of a simple RC electrical circuit, where resistors and capacitors are connected in series. They proposed that this mapping facilitates the quantification of fan-out as well as retroactivity’s understanding. To infer this mapping, they consider promoters as capacitors. The more “capacitors” involved in the system, the more time is needed to charge them all; so the system’s response time slows down. They also consider the observation in Buchler and Cross (2009) that bound promoters act as a reservoir of potentially free transcription factors. When the transcription factor molecules change in number, depending upon the parameters of the system, transcription factor binding to downstream promoters buffers the change in the number of free transcription factor molecules. This buffering slows down transient dynamics.

A work by Jayanthi and Del Vecchio (2012) contrasted the role of loads as retroactivity inducers in a simple system composed of a positive and a negative transcription factor *in silico*. They compared the output concentration of a gene regulated by such an activator and repressor given the following three scenarios: (*i*) the presence of loads regulated by the repressor, (*ii*) the presence of loads regulated by the activator, and (*iii*) the presence of loads in both regulators. The first case, adding loads to the repressor, causes an increase in the oscillatory period of the system. The second one, depending on the amount of loads, turns off system’s ability to oscillate. The third and last case, allowed the authors to tune the system’s oscillation period. These results, along with other previously published theoretical results, promoted the experimental exploration of retroactivity effects *in vitro* and *in vivo*. One of the main features of this work is that the authors suggest that the kinetic rates could be modified by simply adding DNA with binding sites avoiding then to modify promoters or add degradation tags to proteins.

### 3.3 Dynamically insulating synthetic modules for more reliable synthetic systems

**Figure 6:**
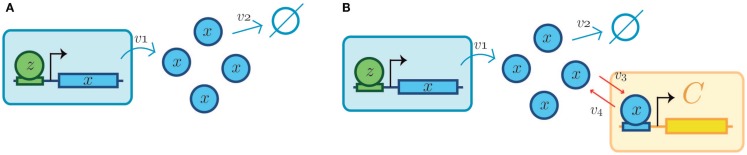
**Retroactivity changes the system’s behavior (focus on retroactivity to the output)**. **(A)** Isolated input–output module (based on Figure 1A). This is a cartoon representation of the system, with the processes involved in the module output in blue arrows and with *v*_1_ denoting the rate of production of *x* (output signal) due to the presence of a transcription factor *z*. *v*_2_ denotes the degradation of *x*. Here, the module in blue would be equivalent to *A* in Figure 1. **(B)** Cartoon representation of the connected system, with the involved processes in the module output denoted by blue arrows and connection processes represented by red arrows as a set of binding/unbinding events (with *v*_3_ and *v*_4_ the association and dissociation rates, respectively). Here, *v*_3_ denotes the loss in *x* species, which changes the upstream’s output concentration and thus its behavior. As mentioned in the main text, the retroactivity, and thus its potential to change behavior, depends on the number of downstream modules and the rates of association and dissociation. Here, the blue module would be equivalent to module *A* in Figure 1B and the yellow one would be analogous to the *B* one in Figure 1B. *v*_3_ and *v*_4_ play the role of *x* and *s*, respectively, in Figure 4C. The schematic representations are based on those used in Del Vecchio et al. and Sauro et al. Further details of how each of this systems aid retroactivity insulation are given in the main text.

The conception of retroactivity as a disruptive signal led to the proposal of dynamic insulation components, which could help in the construction of modularly structured synthetic biomolecular systems [see Franco et al. (2009)]. Two possible dynamic insulation schemes have been proposed. The first one is inspired by the standard modular design of electronic analog devices and comprises the implementation of an insulation device built around a signal amplifier regulated by a negative feedback loop. This dynamic insulation device is based on the well-known operational amplifier (or OPAMP) and has been called amplification-degradation strategy [see Del Vecchio et al. (2008)]. The second dynamic scheme to attain insulation is based on time-scale separation and its role in retroactivity (Figure 6). In what follows, both insulation dynamic strategies are succinctly described.

#### 3.3.1 Strategy Based on Gain Amplification-Degradation

This retroactivity attenuation strategy follows the standard dynamical insulation scheme from analog electronic engineering design. There, to ensure infinite impedance^3^ in the input channel of a downstream device when connecting it to the output channel of an upstream one. Del Vecchio et al. (2008) proposed a dynamic retroactivity-insulation device consisting, as we just mentioned, of the simultaneous amplification and regulated degradation of the output signal (negative feedback) from the upstream transcriptional device. Both amplification and negative feedback gains need to be equally large to maintain the net original signal at the output of the upstream module upon interconnection. Thus, by means of attenuation, retroactivity can be minimized.

To describe how this gain amplification-degradation works in mathematical terms when using a closed loop strategy (i.e., the negative regulation is controlled by the system’s output), let us consider the system *S* in Figure 5C. Where *z* would be the input, *x* the output, and *s* the retroactivity to the output falling on *S*. Here, we could denote the *x* output, as a function of *z* and *s* in the following way:

x=z+s

**Figure 7:**
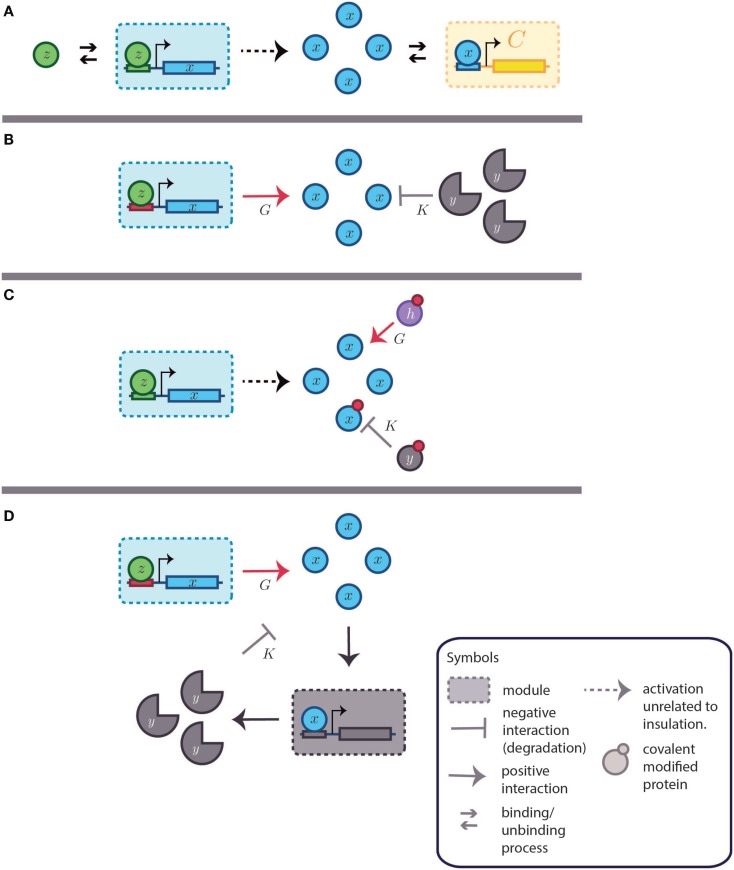
**Dynamic insulation schemes based on both gain amplification-degradation and time-scale separation**. *K* and *G* are the degradation and amplification reactions, respectively. **(A)** Basic system without insulation. A transcription factor (TF), represented by *z*, activates the system [partially omitted in **(B–D)**]. The small arrows represent binding and unbinding interactions of TFs. The modules are delimited by a square with a dashed perimeter. Downstream to the module and regulated by the module’s output is the module *C*. The retroactivity to the output of the first module, the one producing *x*, depends on the association and dissociation rates as well as in the amount of binding sites present in module *C*. Other parameters, as the TF–DNA complex degradation rate also influences the retroactivity value and its effects under the upstream module. The big arrow denotes the output production. The downstream module (in yellow and denoted by *C*) is obviated in the next items for simplicity. As in Figure 5, the blue module can be considered analogous to module *A* and the yellow one is equitable to *B*. **(B)** Gain amplification- degradation. Amplification is attained by using a strong and non-leaky binding site (in red). Degradation is achieved by a protease (*y*). **(C)** Time-scale insulation by covalent modification of the TF of interest. Amplification is given by a protein (*h*) capable of transducing the covalent signal that activates the TF (*x*). Degradation is caused by another protein (*y*), which removes the modification. **(D)** Alternative feedback dependent degradation scheme. *x* promotes the transcription of a degradation agent *y*.

The procedure to reduce *s* would involve a controlled degradation of the output as in Figure 7D. Here, we will denote this degradation as *Kx*. A large gain (*G*) would be required as well. Such gain would influence the output performance. Given the previous changes, the output equation would be:

$$x=G\left(z-\mathrm{Kx}\right)+s,$$

which can be rewritten as:

$$x=\frac{\mathrm{zG}}{1+\mathrm{KG}}+\frac{s}{1+\mathrm{KG}}.$$

As stated in Del Vecchio (2011), it is easy to see that as *G* grows, the system tends to *z*/*K*, which is independent of *s*.

Del Vecchio et al. tested two possible scenarios of experimental realizations *in silico* to illustrate this insulation strategy. The first one recognizes open-loop gain amplification by a strong non-leaky promoter. As far as negative feedback is concerned, it is achieved via strong regulated degradation (see Figures 7B,D in contrast to 7A). The proposal by Del Vecchio et al. on how to achieve such behavior includes the modification of promoters by mutation and direct targeting of the transcription factor, denoted *X*, with a protease named *Y*. A further improvement of this design model includes mRNA dynamics to avoid ignoring delays between the initial input signal and the module state. It is important to note that such delays may be insignificant in bacteria because transcription and translation are coupled. When the gain is large (1000–100 mRNAs/min per complex of *Z* bound to its cognate site), the performance of the original module improves. By contrast, when gains are small, the system execution began to diminish at gains of approximately 10 mRNAs/min per complex of *Z* bound.

In the second realization scenario using a gain amplification-degradation-based insulator (Del Vecchio et al., 2008), the signal is amplified by phosphorylation of an intermediate protein or transcription factor, and negative feedback is achieved by a phosphatase. Further analysis of this design suggested the utilization of time-scale separation as a design strategy for the construction of insulation devices, which is described in the next subsection.

#### 3.3.2 Dynamic Insulation Strategy Based on Time-Scale Module Separation Achieved Through Covalent Modification

As an alternative to the gain amplification-degradation scheme, a time-scale separation component could be included for dynamic retroactivity attenuation (see Figure 7C). It has been proposed to achieve such time-scale separation by means of a reversible covalent modification cycle. In general, covalent modifications tend to be faster than transcription and translation, justifying time-scale separation. To achieve this strategy, the following would be required: a protein that can be covalently modified by the enzyme (i.e., the transcription factor), the production of an enzyme with the ability to add the covalent group to the transcription factor, another enzyme that removes this group, and a binding site specific for the modified transcription factor. In the case of a phosphorylation modification, the properties and capacity for insulation were tested *in silico* in Jayanthi and Del Vecchio (2011). The authors present a model that includes phosphorylation, realizing that steady-state retroactivity effects can be reduced by increasing the amount of total protein that bridges the signal flux. The model considers an enzyme that is modified by an input so it can modify a second element that will dynamically bridge the signal such as a transcription factor activator. The retroactivity effects – visualized in this case as the change in the bandwidth and amplitude of the protein concentration signal – can be mitigated by increasing the amount of enzymes that covalently modify the signal transmitter protein. After a model reduction procedure, further analysis revealed the capacity of a covalent modification to insulate retroactivity effects due to the connection structure and the fast involved rates in this insulation strategy. It would be interesting to perform this same analysis in more realistic models that have not been subjected to reduction as well as in a physical test-bed. A phosphorylation cycle works as a hybrid strategy because phosphorylation can play an amplification role while dephosphorylation may act as negative feedback in a gain amplification-degradation based insulation scheme, as shown in Jayanthi and Del Vecchio (2009). A recent approach to time-scale separation based on contraction theory with bounded attenuation in the design of biochemical networks is discussed in Del Vecchio and Slotine (2011).

### 3.4 Retroactivity and biological noise

In this section, we briefly detail the relation between biological noise and retroactivity. As noted in Kim and Sauro (2011), the autocorrelation time of the output noise signal increases when the output signal regulates a downstream module. This reinforces the previous results that show how retroactivity delays the systems’ responses.

In order to estimate retroactivity by measuring the noise in the expression of a transcription factor, Kim and Sauro (2011) used the involved fan-out as a new metric for retroactivity based on the noise autocorrelation time function^4^ , which is approximately equal to the response time of the deterministic case. They then define a retroactivity measure as follows:

$$R_{s}=\frac{T_{c}-T_{i}}{T_{c}},$$

where *T_c_* is the correlation time in the connected system, and *T_i_* is the correlation time for the isolated system. As long as an adequate frequency is chosen, this approach can be followed considering the total output protein (including complexes) or just the free protein because both are affected by downstream connections. However, they propose to use the total protein concentration partly because the experimental observation is easier. They consider the example of cells carrying fluorescent dyes, assuming that their fluorescence remains unchanged upon binding. They propose a full procedure for retroactivity estimation that allows to quantify the system’s change caused by interconnection in a relatively direct and simple way. As Kim and Sauro conclude, this scheme may be helpful for experimental synthetic biology because it facilitates to test how interconnection will affect the prescribed module’s function using simple measurements. We want to stress that the interconnection effects that take place in a given system tend to be complex and not obvious from the enlisting of system’s participants *per se*. This reinforces the relevance of using mathematical and computational tools for analyzing the possible outcomes of systems interconnection. In Jayanthi and Del Vecchio (2009), the authors consider that a large gain typically amplifies internal noise. After some analysis, they resolved that for systems performing signal processing at high frequency (a range affected by loads) the loads may affect the output signal quality. They concluded that a balance between amplification and noise augmentation must be reached to avoid interfering with the system’s behavior.

In Herath and Del Vecchio (2014), the authors aim to find a way to balance the increase of downstream gene copy number without affecting the upstream system’s behavior. The increase in gene copy number becomes important because the more molecules of a species, the smaller the internal noise related to this specie will be. It would be interesting to analyze how this property has contributed to shape genomes that have suffered multiple duplications (e.g., plants’ genomes). However, the relevance of more than one gene copy remains elusive for strictly haploid organisms and the ones where just one whole genome duplication has taken place (as in yeast) [see, for instance, Kellis et al. (2004)].

We have described here the research around retroactivity issues in the biosynthetic field and now turn to two possible natural insulation strategies. The first one, as the previously mentioned time-scale separation scheme, is based on covalent modification, and the second one on the kinetic insulation phenomenon.

### 3.5 Covalent modification cascades and kinetic insulation

Signaling pathways made of chains of cascades of covalent modification cycles are a major intracellular signaling mechanism. Activated proteins in one cycle promote the activation of the protein in the next link of the chain. Some examples are methylation–demethylation, activation–inactivation of GTP-binding proteins, and phosphorylation–dephosphorylation.

We have already mentioned how small cycles can be used as retroactivity insulating strategies in synthetic systems. In this subsection, we want to emphasize their possible influence in retroactive responses in natural systems.

As noted by Ventura et al. (2008), with an *in silico* model, in these chains, intrinsic negative feedback exerted between each covalent modification cycle and its predecessor emerges naturally. Because of this intrinsic negative feedback, the cascades allow bidirectional propagation, then challenging the supposed unidirectionality of signaling cascades. The emergent negative intrinsic feedback can produce damped temporal oscillations in the chain or create amplified pathway oscillations in the steady states of the cascade. However, Ossareh et al. (2011) established that these covalent modification cascades attenuate retroactivity if they are long enough. Thus, in principle, long signaling cascades offer a mechanism to attenuate retroactivity.

Jiang et al. (2011) used mathematical models and *in vitro* biomolecular systems to propose that retroactivity in transduction networks can be interpreted as load-induced modulation. Moreover, it may balance noise-filtering capabilities and the transduction network’s ability to process high-frequency stimulation, as the presence of downstream targets reduced the bandwidth of the network.

A closely related line of research is the one centered in kinetic insulation. This is a mechanism for achieving pathway specificity in signaling networks that share common components, as shown in Behar et al. (2007). Using an *in silico* model, the authors propose that temporal dynamics can be exploited by cellular biomolecular systems to route the information through a common component while preventing cross-talk. They propose that kinetic insulation could be tested in MAP signaling cascades where component sharing is common. In accordance with the previous statement, two given associated pathways may respond to a given stimulus on different time-scales depending on signals duration, thus preventing retroactivity effects without the need for an amplification-degradation system. Functional modularity is preserved through this mechanism even if the involved signaling network is physically structured in a *quasi*-modular way. Other important means to achieve differential pathway activation from the same signal are cross-inhibition between pathways; spatial localization in the cell; scaffold protein sequestration; and binding specificity. These differential pathway activation cases have been reviewed in Kholodenko et al. (2010).

### 3.6 *In vitro* experimental results strongly suggest functional roles of retroactivity

Returning to our previous discussion from the Section “Modularity,” retroactivity could be considered a homeostatic mechanism contributing to connecting modules within a cell biomolecular network, while inhibiting the coupling of outer elements. Both theoretical and experimental results suggest such homeostatic role for retroactivity. This means that the study of retroactivity has further biological implications than the ones related only to synthetic biology concerns.

One example of the potential regulatory function of retroactivity signals supported by both theoretical and *in vitro* experimental results is given in Jiang et al. (2011), where retroactivity is shown to reduce the extreme sensitivity proper of disconnected modules in covalent modification cycles. Another interesting point in this work, shown both with models and *wet lab* approaches, is that a downstream load could actually make responses faster under certain circumstances. The necessary conditions were that retroactivity moves the system from an ultrasensitive regime to a regime of lower sensitivity response and that the stimulative effector is not saturating for the system. These results provide insights on how loads could have shaped system’s responses throughout evolution, consolidating the module integrity in the whole cell transcriptional network.

It must be noted that enzyme sequestration in covalently modified signaling cascades causes retroactivity even if explicit feedback connections are absent. Given an *in vitro* experimental test-bed of the nitrogen assimilation metabolic pathway in *E. coli*, Ventura et al. (2010) argued that in signal transduction networks, a downstream module may be regulating an upstream one. This, due to the loads, reduces the ultrasensitivity of the system [see Goldbeter and Koshland (1981)]. Here, we consider load effects in terms of retroactivity signaling. This strongly suggests a role of retroactivity as a functional process and not just as a nuisance signal.

### 3.7 *In vivo* experimental approaches denote the potential fragility of transcriptional networks to retroactivity

In Jayanthi et al. (2013), the authors built a synthetic system to test the retroactivity effects caused by loads. This is one of the first *in vivo* demonstrations of the effects that loads alone may have on a transcriptional regulatory system. They tested this by introducing two plasmids in *E. coli*, one carrying the system and another with its cognate binding sites. Jayanthi and Del Vecchio showed that retroactivity effects depend on the ratio between the system plasmid copy number and the loads plasmid one. Their approaches shed light in how to calculate the extent to which a reporter affects the system it assesses. Other interesting aspects that they analyzed were the sign-sensitive delays caused by retroactivity effects on system induction and de-induction (removal of input signal). Induction implied a delay in the system’s response, while the second one showed that the loads made de-induction faster because of the complex degradation. Even when just one system was analyzed, this result poses an interesting point in the discussion of what role can multiple binding sites be playing. In Burger et al. (2010), further discussion on the topic can be found. Finally, two significant approaches to highlight in the Jayanthi et al. paper are how the authors emphasize the relevance of the transient responses (in contrast to the original steady-state approach of retroactivity) and the use of loads as a means to achieve a precise control of the speed of response of a system to its input in synthetic systems is proposed as well.

Asylum from degradation is another potential functional aspect of retroactivity in transcriptional systems, as noted in Burger et al. (2010), in which decoy “non-functional” transcription factor-binding sites are proposed to prevent transcription factor degradation. Furthermore, it has recently been experimentally demonstrated in Lee and Maheshri (2012) – guided by a model similar to the one analyzed in Burger et al. (2010) but considering degradation – that in some cases non-functional binding sites inhibit gene expression, converting graded responses to ultrasensitive ones. This contrast between very similar systems stresses the importance of details in modeling. The aforementioned alteration of the responses’ sensitivity by downstream binding sites has been previously described and is known as inhibitor ultrasensitivity. An early discussion on this topic can be found in Ferrell (1996) and Buchler and Cross (2009).

## 4 Conclusion and Perspectives

When exploring the relationship between retroactivity and biological complexity, we can follow the “quasi-modularity” ideas proposed in Rhodes (2010) while considering the comment in Lauffenburger (2000). Recalling the example of bacterial flagella, it is argued that systems homeostasis is maintained due to significant embedding and intertwining between the components of biological systems. Those are needed to protect simple cell functions from external signals. Lauffenburger clearly stated that “often the machinery assembled to implement control and safety schemes of a core function is more complicated than the machinery of the core itself.” This seems to be in agreement with retroactivity affecting the sensitivity and midpoint of responses, as shown by Ventura et al. (2010), and the report of Ossareh et al. (2011). A downstream load helps to buffer ultrasensitivity. A tempting question that arises from these observations is whether a given module can not only be defined but also actually work in terms of its constitutive minimal elements required for a specific function or as those same minimal elements plus the assistants that help the function to be achieved. The first option leads to an idyllic scenario for synthetic biology as the function of the module would be completely separated from the required regulatory machinery. “Plain functions” – unstable by themselves – and “module connectors” responsible for the regulation of those modules could be characterized and added to the synthetic biology toolbox. Minimalism in this context is thought as a potential measure of complexity, which could be defined in terms of the set of the most basic independent information processors carrying out a particular function. How to implement this complexity measure remains an open question. The role of regulation as a design strategy to fix lower bounds in the complexity of biosynthetic systems deserves to be explored.

As far as modeling tools are concerned, until recently, the description of retroactivity has been preferentially approached by ordinary differential equations. More realistic modeling approaches similar to the ones implemented in Jiang et al. (2011) would be valuable in quantifying the retroactivity effects on the system’s behavior under different operational conditions. In the same train of thought, it would be interesting to apply more intuitive (and experimentally achievable) measurements of retroactivity as the one proposed in Kim and Sauro (2010).

In order to complement what we already exposed about the relationship between retroactivity and the regulation of multiple targets by transcription factors, we can say that to consider a load larger than the output concentration makes sense for network hubs devoted to regulating whole pathways and stress conditions (Luscombe et al., 2004). Nevertheless, as shown in Gama-Castro et al. (2010), many transcription factors are devoted to the regulation of only one or two targets. The relationship between the potential binding sites where the transcription factor could bind versus the sites where it actually binds needs to be further investigated to shed light on the topic. Furthermore, in natural networks, the involved molecules may be not directly monitored and adjusted to a desired concentration level. This is another topic that should be addressed to further understand the role of retroactivity in natural networks [see Springer et al. (2010)].

In terms of insulation strategies, time-scale separation seems to be an attractive alternative to the gain amplification-degradation scheme that involves changing the promoter-binding rate and the degradation one. However, it would be interesting to contrast how well this and other designs would oppose the delays produced by retroactivity in eukaryotes. An example of such a design could be a system capable of inducing the transcription of a parallel transcription factor, which regulates part of the downstream loads.

As far as biological engineering is concerned, the idea of a genetic compiler that can automatically “design” a biosynthetic system given some high-level instructions has been reviewed in Clancy and Voigt (2010). The authors included a list of some of the requirements for the implementation of an accurate automatic designer. Generating libraries of reliable genetic systems and the biophysical methods to connect them requires a deeper understanding of modularity and retroactivity in biomolecular systems, even if the raw pieces for system assembly are planned to be modular (e.g., promoter, RNA, and proteins domains).

We invite the reader to consider the impact of retroactivity in the consolidation of both systems and synthetic biology research. Retroactivity is a property of biomolecular systems, both natural and synthetic. As noted in Del Vecchio (2013), retroactivity’s further consideration and measurement will be crucial for the future design of robust synthetic systems connected to a “chassis.” The context-dependent behavior of biomolecular systems calls for further inquiry into how dynamical networks achieve robustness and resilience in natural systems. As we already discussed, some of these strategies have been studied before [see Sriram et al. (2009) and Benítez and Álvarez Buylla (2010)]. Properties and elements such as redundancy, feedback, and multiple entries contribute to maintain the systems’ functionality. The latter characteristics make biomolecular networks more integrated and less modular (or *quasi*-modular), providing further robustness to fluctuations, change of parameters, and change of conditions. It is debatable whether such elements provide attenuation to retroactive signals for the proper functioning of biomolecular systems. We would like to invite the reader to consider such a possibility and its implications for the integration of functions in natural biomolecular systems.

We conclude with the following statement: modularity’s dynamic-behaviors are starting to be revealed thanks to the development of research lines such as those concerned with the study of kinetic insulation schemes and the elucidation of the effects of retroactivity. Moreover, synthetic biology provides a very useful mixed theoretical–experimental framework to explore possible simplified issues regarding these topics. This “tinkerer” approach may serve to help enrich and organize further inquiry into the comprehension of natural biological systems and provide tools to design cellular complex biosynthetic networks that are free of operational uncertainty. Additionally, synthetic biology can help to test hypotheses derived from systems biology modularity approaches in broader complex networks that are not necessarily biological in nature.

The big picture of the possible mechanisms for achieving robustness and their combination with resilience in the organisms’ context remains to be further explored and understood. In one recent study, Gyorgy and Del Vecchio (2014) proposed a metric for the modules robustness to interconnection. They structured their proposal, expected to be integrated within system’s level studies, as follows: (*i*) a framework to predict interconnected behavior and, (*ii*) a measure for robustness to connection. At this time, it is technologically possible to implement massive parameter measurements that could aid such an integration and global analysis, which doubtlessly would shed light in our understanding of how natural systems use and cope with retroactivity. However its applicability extent is still limited. There is also a lack of studies using “–omics” information to further support the proposals made in small systems without the need of dynamical simulations. This was the common approach at the beginning of retroactivity research, but a new approximation including a whole range of new questions related to the structure and function of natural signaling and regulatory networks remains to emerge. This would doubtlessly shed light in our current understanding of how natural systems use motifs to cope with interconnections or to take advantage of them; how these strategies differ from the ones proposed in the current engineering approaches and how could we adapt the natural strategies that have not been considered to build better synthetic systems.

## 5 Conflict of Interest Statement

The authors declare that the research was conducted in the absence of any commercial or financial relationships that could be construed as a potential conflict of interest.

## Footnotes

^1^By “controllability of a non-isolated system,” we mean its structural property that allows external stimuli to drive the system from a given internal dynamical steady state to another one. Then, structural controllability depends on the way the exogenous information flows to the system, *i.e*., if the pattern of connectivity of the system with its surroundings changes, its controllability properties will change as well. Steady state transitions in an isolated, *i.e*., structuraly noncontrollable, system can occur as a consequence of stochastic biomolecular fluctuations under the dynamical constraints imposed by the underlying epigenetic landscape, as described in (Álvarez Buylla et al., 2008).

^2^A *mini-module* is the simplest dynamical element (*i.e*., an elementary memory-based automaton) with an individual input and an individual output that can be identified in a given dynamical network. In the context of John Rhodes ideas, exposed in (Rhodes, 2010), the concept of mini-module would correspond to the elementary dynamical system acting as the basic constructive block of complex dynamical networks. For instance, if a network of biochemical reactions is described in (finite-state) discrete-time terms, a mini-module would correspond to the simplest memory-based automaton, *i.e*., a simple fixed-time delay. In a finite-dimensional continuous-time description case, a mini-module would correspond then to a mathematical integrator. John Rhodes comes to analyze the complexity of dynamical networks in algebraic terms. He studies how feedback-based interconnections reduce the length of dynamical chains, and then the associated complexity of a given chain of biochemical reactions. An example of a mini-module in transcriptional networks could be a gene regulated by a transcription factor (input) whose transcription triggers the production of a protein (output), bypassing the stages of transcription and post-transcriptional modifications.

^3^In electrical and electronic engineering, an electrical load is said to display infinite impedance at its input upon interconnection to a voltage source, if the given voltage level provided by the source will not drop at all due to the divider effect.

^4^The characteristic time-scale of a random process is the correlation time. This characterizes the time-scale for a time correlation function to decay to zero. Another way to state the correlation time is as the decay time from the gene expression autocorrelation function. In (Kim and Sauro, 2011) an autocorrelation function quantifies the correlation of the signal with itself for a given time-lag.
